# The Impact of Social Role Identity on Communication in Hospital-Based Antimicrobial Stewardship

**DOI:** 10.1017/ash.2021.25

**Published:** 2021-07-29

**Authors:** Julia Szymczak, Brandi Muller, Nikitha Shakamuri, Keith Hamilton, Elizabeth Dodds Ashley, Hilary Babcock, Rebekah Moehring, Jason Newland, Ebbing Lautenbach

## Abstract

**Background:** Evidence-based hospital antimicrobial stewardship interventions, such as postprescription review with feedback, prior authorization, and handshake stewardship, involve communication between stewards and frontline prescribers. Hierarchy, asymmetric responsibility, prescribing etiquette, and autonomy can obstruct high-quality communication in stewardship. Little is known about the strategies that stewards use to overcome these barriers. The objective of this study was to identify how stewards navigate communication challenges when interacting with prescribers. **Methods:** We conducted semistructured interviews with antimicrobial stewards recruited from hospitals across the United States. Interviews were audio recorded, transcribed, and analyzed using a flexible coding approach and the framework method. Social identity theory and role theory were used to interpret framework matrices. **Results:** Interviews were conducted with 58 antimicrobial stewards (25 physicians and 33 pharmacists) from 10 hospitals (4 academic medical centers, 4 community hospitals, and 2 children’s hospitals). Respondents who felt empowered in their interactions with prescribers explicitly adopted a social identity that conceptualized stewards and prescribers as being on the “same team” with shared goals (in-group orientation). Drawing on the meaning conferred via this social role identity, respondents engaged in communication strategies to build and maintain common bonds with prescribers. These strategies included moderating language to minimize defensive recommendations when delivering stewardship recommendations, aligning the goals of stewardship with the goals of the clinical team, communicating with prescribers about things other than stewardship, compromising for the sake of future interactions, and engaging in strategic face-to-face interaction. Respondents who felt less empowered in their interactions thought of themselves as outsiders to the clinical team and experienced a heightened sense of “us versus them” mentality with the perception that stewards primarily serve a gate-keeping function (ie, outgroup orientation). These respondents expressed deference to hierarchy, a reluctance to engage in face-to-face interaction, a feeling of cynicism about the impact of stewardship, and a sense of low professional accomplishment within the role. Respondents who exhibited an in-group orientation were more likely than those who did not to describe the positive impact of stewardship mentors or colleagues on their social role identity. **Conclusions:** The way antimicrobial stewards perceive their role and identity within the social context of their healthcare organization influences how they approach communication with prescribers. Social role identity in stewardship is shaped by the influence of mentors and colleagues, indicating the importance of supportive relationships for the development of steward skill and confidence.

**Funding:** No

**Disclosures:** None

